# Intake of ultra-processed food, dietary diversity and the risk of nutritional inadequacy among adults in India

**DOI:** 10.1017/S1368980023002112

**Published:** 2023-12

**Authors:** Srishti Mediratta, Santu Ghosh, Pulkit Mathur

**Affiliations:** 1 Department of Food and Nutrition and Food Technology, Lady Irwin College, University of Delhi, New Delhi, India; 2 Department of Biostatistics, St John’s Medical College, Bangalore, India; 3 Department of Food and Nutrition and Food Technology, Lady Irwin College, University of Delhi, Sikandra Road, Mandi House, New Delhi, 110001, India

**Keywords:** Diet quality, Adequacy, Micronutrient, Risk assessment

## Abstract

**Objective:**

This study assessed diet diversity and consumption of ultra-processed foods and explored its impact on macronutrient intake and risk of micronutrient inadequacy.

**Design:**

Cross-sectional, non-probability snowball sampling.

**Setting:**

Nutrient intake was assessed using 24-h dietary recall method and diet diversity through FAO-diet diversity score (DDS). Mann–Whitney *U* test was used to assess differences in risk of inadequacy across gender. Spearman’s rank correlation assessed associations between energy contributed by ultra-processed food and risk of nutrient inadequacy.

**Participants:**

A total of 589 adults (20–40 years) belonging to upper-middle and high-income groups.

**Results:**

The average individual DDS was 4·4 ± 0·6. Most of the participants (>80 %) had intakes less than national recommendations of pulses/eggs/flesh foods, milk/milk products, fruits, vegetables and nuts. Ultra-processed foods contributed to 17 % of total energy intake, 12 % of protein, 17 % of carbohydrate, 29 % of added sugar, 20 % of total fat and 33 % of Na intake. The average risk of nutrient inadequacies for Zn (98 % *v*. 75 %), folate (67 % *v*. 22 %) and niacin (83 % *v*. 44 %) was higher among males than females (*P* < 0·001). The average risk of nutrient inadequacies for Fe (58 % *v*. 7 %), vitamin B_6_ (95 % *v*. 90 %) and vitamin A (68 % *v*. 44 %) was higher among females than males (*P* < 0·001). There was a positive correlation between energy contributed by ultra-processed food and risk of niacin (*ρ* = 0·136, *P* = 0·001) and folate (*ρ* = 0·089, *P* = 0·049) inadequacy.

**Conclusion:**

Reformulating ultra-processed food to reduce fat, sugar and salt and increase micronutrients and behaviour change communication strategies that promote dietary diversity will improve micronutrient adequacy and diet quality.

In India, the prevalence of obesity and diet-related non-communicable diseases such as diabetes and hypertension has risen in the past 5 years^(1)^. Overconsumption of energy-dense and nutrient-poor diets is one of the major factors that increase the risk of development of such diseases^(2)^. Many adults prefer ultra-processed foods that are energy dense, high in fat, sugar and salt and low in nutrient content^(3)^.

The NOVA food classification system groups foods into unprocessed or minimally processed foods (such as fresh fruits and vegetables, grains, milk, eggs), processed foods that are made by adding processed culinary ingredients like salt, oil, butter or sugar to foods (such as canned vegetables and legumes, freshly made unpackaged breads and cheeses) and ultra-processed foods that are made by industrial processes using preservatives, additives, synthetic flavour enhancers in order to enhance the taste, texture, shelf life and convenience (such as ready to consume packaged products like carbonated soft drinks, snacks, chocolate, confectionary, ice cream, breads, spreads, biscuits, cakes, breakfast cereals, fruit drinks, pre-prepared ready to heat foods, instant soups, noodles and desserts)^(4)^. An increased consumption of ultra-processed foods can cause many health challenges such as abdominal obesity, dyslipidaemias, high blood pressure and hyperglycaemia to the population if diets are not modified^(5)^. A study conducted in India showed that the consumption of ultra-processed food has reached all socio-economic segments of the society^(6)^. The main drivers towards the consumption of packaged ultra-processed food among adults are busy schedule, lack of time to prepare cooked meals, irregular work hours and lack of willpower to resist tasty food and adopt healthy eating practices^(7)^.

India is in a state of ‘triple burden of malnutrition’ with rise in diet-related non-communicable diseases and micronutrient deficiencies along with undernutrition^(8)^. Studies have shown that micronutrient deficiencies arise due to inadequate intake of foods and nutrients in the diet. The universally standardised FAO-diet diversity score (FAO-DDS) is a tool often used to assess diet quality using categories of food groups^(9)^. In India, a majority of the population have dietary intake of vegetables (83 %) and fruits (71 %) lower than the MyPlate Indian Council of Medical Research – National Institute of Nutrition (ICMR-NIN) recommendations^(10)^. A study showed that adults in India had greater than 80 % risk of deficiencies of Ca, vitamin A and folate in the diet^(11)^. The national survey also showed that the average nutrient intake of protein, Ca, Fe, thiamine, riboflavin, niacin and vitamin A was below the recommendations among urban adult population^(12)^. Assessing the present dietary situation is a step towards addressing nutrient deficiencies to improve the public health^(13)^. This study aims to assess the contribution of ultra-processed foods to macronutrient intake and the risk of micronutrient inadequacy in the diet.

## Methods

### Study design

The study had a cross-sectional survey design with non-probability purposive sampling. A multistage non-probability sampling was used, whereby residential areas were selected from each of the four geographical zones of the city by purposive sampling and participants in each selected residential area were selected by snowball sampling. The city of Delhi was geographically divided into four zones: north, south, east and west. A total of twenty-three residential areas from four geographical zones of the city, north (6), south (6), east (5) and west (6), were purposively selected depending on the ease of accessibility and permission from resident welfare associations. Residential areas representing the upper-middle and high-income groups were included based on the categorisation given by the city’s municipal corporation^(14)^. A total of 589 adults in the age group 20–40 years belonging to upper-middle-income group and high-income group were selected, who engaged in the purchase of packaged and ultra-processed food items. We purposively looked at those in the higher income groups because we wanted to explore dietary consumption when there are no financial constraints. Resident welfare associations of residential areas were contacted, and key informants were identified, who helped to identify households where further sample size of the study could be completed. One participant was selected from the identified household and thereafter snowball sampling was applied. Selection of participants depended on the family income also and not solely on the location of the house. The Modified Kuppuswamy’s Socio-economic Scale^(15)^ was applied to categorise participants into, that is, upper-middle-income and high-income groups.

This study was part of a wider research work where the sample size was calculated on the basis of proportion of adults with a low fruit and vegetable consumption, because it is one of the main indicators of diet quality. According to the WHO guidelines, a low fruit and vegetable consumption is defined as consuming less than five servings of fruits and/or vegetables per day^(16)^. A study reported the proportion of adults with low fruits and vegetable consumption to be 74·4 % among males and 74 % among females^(17)^ in India. The sample size for males and females was calculated separately with a CI of 95 % and confidence limits set at 5 %. Assuming a dropout rate of around 10 %, around 684 participants were enrolled in the study; after an attrition of about 14 %, data collection was stopped when 589 participants were enrolled from the four geographical zones of the city based on the inclusion criteria. The sample size estimation is provided in supplemental file (Table 1).

**Table 1:** Proportion of individuals consuming different food groups (*n* 589)

Food groups	Total		Male		Female	
	*n*	%	*n*	%	*n*	%
Starchy staples	589	100	293	100	296	100
Dark green leafy vegetables	69	12	42	14	27	9
Other vitamin A-rich fruits and vegetables	15	2·5	10	4	5	2
Other fruits and vegetables	589	100	293	100	296	100
Organ meat	5	1	2	1	3	1
Meat and fish	131	22	69	24	62	21
Eggs	91	15	46	16	45	15
Legumes, nuts and seeds	589	100	293	100	296	100
Milk and milk products	564	95	287	98	277	94

Note: All responses are presented as number (percentage) of participants.

### Data collection

The dietary and nutrient intakes were assessed using the 24-h dietary recall method repeated on two non-consecutive days (one working and one non-working). Participants were asked to recall all the food items consumed starting from morning till night over the past 24 h. They were probed on the method of food preparation such as consistency like thin/thick, greasiness of food item and ingredients used in preparation. The amount of food items consumed was recorded by using two- and three-dimensional food models of standardised spoons, ladles, bowls, plates and glass. A calibrated electronic digital weighing scale was used for standardising the amounts of food stuffs consumed by respondents as reported in the 24-h recalls. In the case of ultra-processed packaged food products, nutrient content was taken from the respective food labels and, in the case of composite food dishes, standardised recipes were used for recording ingredients to capture the nutrient contents of dishes^(18,19)^.

#### Assessing dietary diversity and food group adequacy

The FAO-individual dietary diversity score (DDS), which has a standardised questionnaire for universal application, was used to assess dietary diversity in the diets of participants. For the purpose of scoring, the foods are classified into nine food groups, such as starchy staples, dark green leafy vegetables, other vitamin A-rich fruits and vegetables, other fruits and vegetables, organ meat, meat and fish, eggs, legumes nuts and seeds, milk and milk products. A score of 1 is given for each food group consumed over the past 24 h. The scores were then added to arrive at a final score. There are no established cut-off points, in terms of number of food groups to indicate adequate dietary diversity in the DDS. However, it is recommended to use the mean scores or distribution of scores. Hence, the scores were divided based on tertiles as less than or equal to 4, 5–6 and above 6^(9)^. Food group intake adequacy was assessed on the basis of the ICMR-NIN MyPlate recommendations^(10)^. ‘There were no significant differences in the DDS and adequacy of food groups based on income group of the participants’.

#### Measuring risk of nutrient inadequacy

First, the individual risk of inadequate intake was estimated based on the probability that the requirement is greater than the intake. The population distribution of the requirement of each nutrient was derived from reported estimated average requirement (EAR), RDA and type of distribution by ICMR. In case of symmetric requirement distribution, normal probability distribution with mean at *µ* = EAR and sd at *σ* = (RDA-EAR)/1·96 was assumed. For a positively skewed distribution, lognormal probability distribution with scale and shape parameters as *µ* = log (EAR) and *σ* = {log (RDA)-log (EAR)}/1·96 was assumed, respectively. Therefore, the individual risk of inadequate intake of a given intake *x* was derived as in the case of normal distribution as:

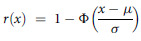




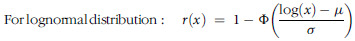

where 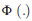 is the cumulative distribution function of standard normal distribution.

### Statistical analysis

The period of data collection was from March 2019 to February 2020. The 24-h dietary intakes were entered in ‘DietCal’ software version 9.0 (Profound Tech Solutions, 2014), and the nutrient intakes were analysed. The software utilises the nutritive values of food items from the Indian Food Composition Tables^(20)^. The contribution of ultra-processed foods to the daily macronutrient intakes was expressed as percentage. Statistical software R version 4.2.1 (R core Team, 2022) was used to analyse the risk of nutrient inadequacies in the diet (see online Supplemental file, Table 2). The average risk of inadequate intakes with 95 % confidence intervals was reported for each nutrient. Mann–Whitney *U* test was used to assess the difference in probability of nutrient inadequacy (average risk) across gender. The association between energy contributed by ultra-processed food and risk of micronutrient inadequacy was calculated by Spearman’s rank correlation. Statistical significance was assumed at 5 % level (*P* < 0·05).

**Table 2:** Diet diversity scores (DDS) among participants (*n* 589)

Variables	DDS		Mann–Whitney *U* test
Total (*n* 589)	4	4,5	
	Median	IQR	*P* value
Gender			
Male (*n* 293)	5·3	4·4–6·2	**0·008** **
Female (*n* 296)	4·8	4·3–5·7	
Age			
20–30 years (*n* 452)	4·5	3·5–4·6	0·88
30–40 years (*n* 137)	4·6	3·7–4·5	
Marital status			
Married (*n* 129)	4·6	3·1–4·8	0·77
Unmarried (*n* 460)	4·4	3·5–4·6	
Eating habit			
Vegetarians (*n* 382)	4·5	4·1–4·8	<**0·001** **
Non-vegetarians (*n* 207)	5·4	4·3–6·4	

** Significant at *P* < 0·01.

## Results

The sample (*n* 589) comprised of equal proportion of males (50 %) and females (50 %). Participants were divided into upper-middle-income group (*n* 118) and high-income group (*n* 471).

### Diet diversity scores

Table 1 shows that all participants consumed starchy staples, other fruits and vegetables and legumes, nuts and seeds. Milk and milk products were consumed by most (95 %) of the participants. Few participants consumed meat and fish (22 %), eggs (15 %) and dark green leafy vegetables (12 %). Very few of the participants consumed foods from the other vitamin A-rich fruits and vegetables (2·5 %) and organ meats (1 %). A higher percentage of male participants consumed dark green leafy vegetables (14 % *v*. 9 %), meat and fish (24 % *v*. 21 %) and milk and milk products (98 % *v*. 94 %) compared with females. The average individual DDS among participants was 4·4 ± 0·6, and the range was (3–6) out of a total possible score of 9. Most of the (58 %) participants had DDS in the first tertile consuming only four food groups, 33 % of the participants had scores in the second tertile consuming five food groups and only 9 % had scores in the highest tertile consuming more than six food groups out of the nine food groups. Table 2 shows that the differences in the individual DDS across socio-demographic variables were significant for gender (*P* = 0·008) and eating habits (*P* < 0·001). Males and non-vegetarians had a higher dietary diversity score compared with females and vegetarians, respectively.

### Food group adequacy

Table 3 shows the intake of food groups by participants in comparison with the ICMR-NIN MyPlate recommendations. A majority of participants had dietary intake of cereals (71 %), total fat (83 %) and other foods such as chips, chocolate and packaged foods (86 %) more than the MyPlate recommendations. The intake of most of the participants was less than the MyPlate recommendations for food groups like pulses/eggs/flesh foods (98 %), milk and milk products (90 %), fruits (93 %), vegetables (80 %) and nuts (92 %). The intake of cereals (37 % *v*. 21 %, *P* < 0·001) and other foods (18 % *v*. 10 %, *P* = 0·005) was within the recommended intakes of a higher proportion of females. A higher percentage of males consumed pulses/eggs/flesh foods (*P* = 0·03) within the recommended range. A higher percentage of participants belonging to high-income group consumed milk and milk products, vegetables and fruits above the recommendations compared with those belonging to upper middle income. A higher percentage of participants belonging to upper-middle-income group consumed food from other groups within the recommended range compared with those belonging to high income.

**Table 3:** Percentage adequacy of dietary intake among participants (*n* 589) based on ICMR-NIN MyPlate recommendations

Food groups			Gender				Chi-square test
	Total (*n* 589)		Male (*n* 293)		Female (*n* 296)		
	*n* consuming recommended intakes	%	*n* consuming recommended intakes	%	*n*	%	
Cereals (≤40 % E)	173	29	63	21	110	37	*χ*^2^ = 17·408, *P* ** < 0·001** **
Pulses/eggs/flesh foods (≥17 % E)	11	2	45	15	39	13	*χ*^2^ = 4·467, *P* = **0·03** *
Total fat (20–30 % E)	98	17	57	20	41	14	*χ*^2^ = 3·332, *P* = 0·06
Milk and milk products (≥300 ml/g)	61	10	26	9	35	12	*χ*^2^ = 1·525, *P* = 0·21
Vegetables (≥300 g)	115	20	62	21	53	18	*χ*^2^ = 0·993, *P* = 0·31
Fruits (≥75 g)	41	7	14	5	27	9	*χ*^2^ = 3·065, *P *= 0·08
Nuts (≥30 g)	49	8	48	16	41	14	*χ*^2^ = 0, *P*-value = 0·98
Others (<10 % of E)	80	14	28	10	52	18	*χ*^2^ = 8·051, *P* = **0·005** *

* Significant at *P* < 0·05,

** Significant at *P* < 0·01.

Other foods include chips, chocolate and packaged foods.

Eggs/Flesh foods are consumed as alternatives to pulses in Indian diets.

### Contribution of ultra-processed food to nutrient intake

According to the NOVA classification, the ultra-processed food items were categorised into the following categories that are packaged and mass produced, that is, beverages (carbonated, milk based, fruit based), savoury snacks (namkeen, chips), confectioneries, chocolate, ice cream, breads, biscuit/cookies, cake/pastries, breakfast cereals, noodles, pasta, processed meat products (ham, sausage, seekh), ready to eat dishes (Indian gravies and pulses), ready to cook (instant soup, frozen vegetarian snacks, frozen non-vegetarian snacks) and spreads/dressings.

Macronutrient contribution to daily energy intake by fat, protein and carbohydrate was 33 %, 9·5 % and 56 %, respectively. The amount of the ultra-processed foods consumed are presented in Table 4. Based on the consumption as recorded during the 24-h recall, 47 % of the participants consumed biscuits, 20 % namkeens (savoury snack), 19 % spreads and dressings, 19 % chips and 14 % breads. Rest of the foods were consumed by less than 10 % of the participants. Table 5 shows that ultra-processed foods contributed to 17 % of total energy intake, 12 % of protein, 17 % of carbohydrate, 29 % of added sugar, 20 % of total fat and 33 % of Na intake in the diet. Male participants had a significantly higher energy (*P* < 0·001), protein (*P* = 0·014), total fat (*P* < 0·001), carbohydrate (*P* < 0·001) and free sugar (*P* < 0·001) intake from ultra-processed foods in the diet compared with females.

**Table 4:** Consumption of ultra-processed food items (g/ml) among participants (*n* 589)/d

Food categories	*n*	%	Mean (g/ml)	SD	Range (min–max)	Median	IQR
Spreads and dressing	111	19	11	5	5–20	10	10–15
Breads	85	14	61	15	30–90	60	60–70
Namkeen (savoury snack)	119	20	37	10	10–80	40	30–45
Biscuits	274	47	20	7	10–70	20	20–20
Chips	113	19	43	12	20–90	40	40–50
Breakfast cereal	23	4	47	12	20–80	40	40–50
Ice creams	56	10	49	14	10–90	50	40–60
Chocolates	15	3	17	5	10–20	20	10–20
Cakes	54	9	28	11	10–90	30	20–30
Carbonated beverage	52	9	280	93	100–500	300	200–350
Milk-based beverage	40	7	215	57	80–400	200	200–200
Fruit juice	22	4	232	41	160–360	220	220–240
Noodles	48	8	59	12	30–80	60	50–60
Pasta	35	6	70	14	50–90	60	60–90
Processed meat products	31	5	33	13	15–70	30	25–40
Ready to eat	25	4	90	41	40–160	80	48–120
Instant soup	26	4	7	5	2–23	5	3–10
Frozen vegetarian snacks	52	9	37	11	15–60	32	32–40
Frozen non-vegetarian snacks	58	10	49	11	5–20	45	45–60

Note: These foods were packaged, and the list of ingredients was checked to classify them as ultra-processed. All responses are presented as number (percentage) of participants.

**Table 5:** Dietary intake and contribution of ultra-processed foods to the diet

Macronutrient	Total (*n* 589)				Male (*n* 293)		Female (*n* 296)		Mann–Whitney *U* test *P* value	% nutrient contribution		
	Mean	sd	Median	IQR	Median	IQR	Median	IQR		Total (*n* 589)	Male (*n* 293)	Female (*n* 296)
Energy, kcal	383	146	374	272–465	413	337–487	316	240–422	**<0·01** **	17	18	17
Total fat, g	16	7	15	10–23	18	12–23	12	10–20	**<0·01** **	20	22	20
Carbohydrate, g	52	23	50	36–65	52	40–67	45	30–60	**<0·01** **	17	17	17
Protein, g	6·4	3·3	6	4–8	6	4·5–7·9	5·4	3·1–8·3	**0·014** *	12	11	13
Free sugar, g	17	12	13	9–19	15	10–21	10	8–16	**<0·01** **	29	32	24
Na, mg	266	50	123	51–343	126	51–350	100	51–331	0·85	33	26	29

* Significant at *P* < 0·05,

** Significant at *P* < 0·01.

Note: Percent (%) contribution = nutrient intake from ultra-processed foods/total nutrient intake.

IQR: interquartile range, that is, P_25_–P_75_.

### Risk of nutrient inadequacies

Nutrient adequacy was analysed by applying the EAR cut point method and by the probability approach (Table 6). Most of the participants had nutrient intakes of Ca (97 %), Zn (98 %), riboflavin (100 %), niacin (68 %), vitamin B_6_ (96 %), folate (43 %) and vitamin A (60 %) lower than the EAR. The nutrient requirements of Fe, folate and vitamin A are not normally distributed and log values were taken in these cases. The average risk of inadequate intake was considered as the estimate of probability of inadequate intake of the population^(21)^. The nutrient intake was considered adequate if the probability of nutrient inadequacy was less than or equal to 50 %^(22)^.

**Table 6:** Average risk of nutrient inadequacy among adults (*n* 589)

Nutrients			Intake				Requirements				Risk of inadequacy				*P* value
	Total (*n* 589)		Male		Female		Male		Female						
	<EAR		Mean	sd	Mean	sd	EAR	RDA	EAR	RDA	Males (*n* 293)		Females (*n* 296)		
											Mean	95 % CI	Mean	95 % CI	
Ca (mg)	572	97	425	137	423	164	800	1000	800	1000	0·9575	0·94, 0·97	0·977	0·96, 0·98	<0·001**
Mg (mg)	11	2	463	68	411	64	320	385	270	325	0·029	0,1	0·021	0, 1	**<0·001** **
Fe (mg)	218	37	16	2	14	3	11	19	15	29	0·065	0, 0·28	0·5887	0·2, 0·9	**<0·001** **
Zn (mg)	581	98	10	1	9	1	14	17	11	13·2	0·980	0, 1	0·7543	0, 1	**<0·001** **
Thiamine B_1_ mg)	93	16	1·4	0·2	1·2	0·1	1·2	1·4	1·1	1·4	0·156	0, 0·6	0·2468	0, 0·9	**<0·001** **
Riboflavin B_2_ (mg)	589	100	0·5	0·1	0·5	0·1	1·6	2	1·6	1·9	0·999	0, 1	0·9999	0·9, 1	0·91
Niacin B_3_ (mg)	403	68	10	2	9	2	12	14	9	11	0·831	0, 1	0·4459	0, 1	**<0·001** **
Vitamin B_6_ (mg)	570	96	1·2	0·1	1·1	0·1	1·6	1·9	1·6	1·9	0·906	0·55, 1	0·9531	0·7, 1	**<0·001** **
Folate B_9_ (µg)	255	43	231	40	220	50	250	300	180	220	0·675	0,1	0·2203	0, 1	**<0·001** **
Vitamin C (mg)	27	5	92	23	90	25	65	80	55	65	0·117	0, 0·7	0·0522	0, 1	**<0·001** **
Vitamin A (µg)	353	60	503	110	330	191	460	1000	390	840	0·437	0·1, 0·77	0·680	0·1, 1	**<0·001** **

** Significant at *P* < 0·01.

The average risk of nutrient inadequacies for Zn, niacin and folate was higher among males than females (*P* < 0·001). The average intake of niacin was (10 ± 2) mg/d in males and (9 ± 2) mg/d in females, while the EAR is 12 mg/d for males and 9 mg/d for females and the average risk of niacin inadequacy among males was 83 % and 44 % among females, so the dietary niacin was inadequate for males and not for females (*P* < 0·001). The average intake of folate was (231 ± 40) µg/d in males and (220 ± 50) µg/d in females, while the EAR is 250 µg/d for males and 180 µg/d for females and the average risk of folate inadequacy among males was 67 % and 22 % among females so the dietary folate was inadequate for males and not for females (*P* < 0·001).

The average risk of nutrient inadequacies for Ca (97 % *v*. 95 %), Fe (58 % *v*. 7 %), vitamin B_6_ (95 % *v*. 90 %) and vitamin A (68 % *v*. 44 %) was higher among females than males (*P* < 0·001). The average intake of vitamin A was (503 ± 110) µg/d in males and (330 ± 191) µg/d in females, while the EAR is 460 µg/d for males and 390 µg/d for females and the average risk of vitamin A inadequacy among males was 44 % and 68 % among females so the dietary vitamin A was inadequate for females and not for males (*P* < 0·001).

The average risk of nutrient inadequacies for Mg, thiamine B_1_ and vitamin C was below 50 % for both genders, so the diets were more than adequate for these nutrients. The average risk of nutrient inadequacy for riboflavin among participants was 99 % and their diets were highly inadequate in these nutrients. There were significant associations (*P* < 0·05) between the risk of nutrient inadequacy and the percentage energy contributed by ultra-processed food (Table 7). There was weak negative correlation between the percentage energy contributed by ultra-processed food and the risk of Fe (*ρ* = −0·08, *P* < 0·05), vitamin A (*ρ* = −0·084, *P* = 0·041) and riboflavin (*ρ* = −0·152, *P* < 0·001) inadequacy. There was a positive correlation between energy contributed by ultra-processed food and the risk of niacin (*ρ* = 0·136, *P* = 0·001) and folate (*ρ* = 0·089, *P* = 0·049) inadequacy.

**Table 7:** Association between percentage energy contributed by ultra-processed food and the individual risk of nutrient inadequacy

Nutrients	Spearman’s rho (*ρ*)	*P* value
Ca (mg)	0·044	0·29
Mg (mg)	0·019	0·64
Fe (mg)	−0·088	**0·03** *
Zn (mg)	0·007	0·86
Thiamine B_1_ mg)	−0·078	0·05
Riboflavin B_2_ (mg)	−0·152	**<0·001** **
Niacin B_3_ (mg)	0·136	**<0·001** **
Vitamin B_6_ (mg)	−0·062	0·13
Folate B_9_ (µg)	0·081	**0·049** *
Vitamin C (mg)	−0·036	0·38
Vitamin A (µg)	−0·084	**0·04** *

Note:

* significant at *P* < 0·05,

** significant at *P* < 0·01.

## Discussion

In the present study, the mean dietary diversity score could only reach half of the total possible score. The major food groups missing from diets included meat and fish, eggs, vitamin A-rich fruits and vegetables and dark green leafy vegetables. However, it was heartening to see that all respondents were consuming legumes, nuts and seeds that are rich sources of protein, good fats, fibre and several vitamins and minerals. Legumes have been associated with several health benefits such as improved gut functioning, lowered risk of diabetes, heart diseases, hypertension and overweight^(23)^. Compared with the ICMR-NIN MyPlate recommendations, the majority of the participants (>80 %) had dietary intake of pulses/eggs/flesh foods, milk and milk products, fruits, vegetables and nuts below the recommendations. These protective food groups are hence not being consumed in adequate quantities to derive their health benefits. Milk and milk products are the most bioavailable form of Ca, which is important for bone health. Fruits and vegetables are a store house of vitamins and minerals and also fibre, which protects against several diet-related non-communicable diseases. Similar to our results, a study conducted in Kolkata, India showed that less than half of the respondents consumed dark green leafy vegetables (45 %), vitamin A-rich fruits and vegetables (38 %), organ meats (35 %) and eggs (12 %) even in urban, high-income areas^(24)^. In another study conducted in China, the mean DDS score was 5·2 ± 1·1, but the intake of vegetables, fruits and eggs was lower as per the recommendations^(25)^. Most (82 %) of the participants had total fat intake more than 30 % of total energy per day and 18 % had total fat intake between 20 % and 30 % of total energy per day. None of the respondents had total fat intake less than 20 % of the total energy per day, while some (20 %) participants consumed more than 35 energy %. Most (65·5 %) of the participants consumed more than 10 % of total energy per day from added sugars.

The ‘MyPlate for the Day’ developed by the ICMR-NIN has been designed on the basis of RDA, which typically illustrates proportion of foods from different food groups to be sourced for a 2000 kcal Indian diet. In the present research, male participants and those who had non-vegetarian eating habit had a higher DDS. In comparison with the ICMR-NIN MyPlate recommendations, a higher percentage of males had dietary intake from pulses/eggs/flesh foods (*P* = 0·03) within the recommended intakes compared with females. A study conducted among adults showed that those with low intake of non-vegetarian foods have lower DDS with higher nutrient inadequacies^(26)^. Generally, women opt for vegetarian foods due to the personal beliefs, consciousness towards body image and a feeling of aversion towards meat products^(27)^. Also, a higher percentage of participants from the high-income group had food group adequacy from pulses/eggs/flesh foods, milk and milk products, vegetables and fruits compared with those belonging to upper-middle income. However, a higher percentage of participants from upper-middle-income group consumed food from others group within the recommended range compared with those belonging to high income. Participants from the income groups studied were not constrained by the price of food products. This shows that belonging to a higher socio-economic income group does not always ensure a healthy diet.

In a study conducted in Philippines, rise in socio-economic income led to increased intake of beneficial foods such as milk and chicken in the diet. However, it also resulted in an increased consumption of savoury snacks, ice creams, processed meat and sugar-sweetened beverages among adolescents^(28)^. A study showed that an improved socio-economic condition could lead to increased access and affordability to food items, but choosing healthier food options requires knowledge and understanding of healthy diet^(29)^. A study conducted using dataset from the Indian NFHS 4 survey showed that women in the highest income quintile (OR = 3·24, *P* < 0·001) and those who had high dietary diversity (OR = 1·21, *P* < 0·001) were more likely to be overweight and obese. Increased intakes of ultra-processed foods could be one of the potential causes as they tend to be high in fat, sugar and salt^(30)^.

Results in this study showed that the majority of participants had the risk of micronutrient inadequacies in the diet. The risk of inadequate nutrient intake is defined as the area under the requirement distribution curve that is above the intake, where 50^th^ percentile is the EAR and 97·5^th^ percentile is the RDA^(31)^. Micronutrient inadequacies lead to deficiencies, which cause impaired immune function and poor metabolic and cognitive functions in the human body^(32)^. In a study conducted in India, the mean probability of adequacy for nutrients such as thiamine, riboflavin, niacin, Zn, Fe, vitamin A, vitamin C and Ca was only 37 %, which meant that the diet was inadequate^(33)^.

Creating awareness about dietary diversity and increasing intake of the healthier food groups is a cost-effective way of improving diet quality^(34)^. Nutrition education is a cost-effective strategy that has shown to increase the dietary diversity by 3 % within the household and 9 % increase in the dietary diversity in women in Africa^(35)^. In an intervention study in Bangladesh, where interpersonal counselling and community awareness were utilised to improve diets, showed that the purchase of eggs, flesh foods increased in the intervention group (*P* < 0·01) while purchase of packaged juices, carbonated beverages reduced (*P* < 0·01) as compared to the baseline^(36)^. Food synergy such as including Fe-rich foods along with vitamin C-rich foods in the diet will help to improve the bioavailability of micronutrients^(37)^.

In the present study, the consumption of ultra-processed foods was majorly from food categories such as beverages, biscuits, pasta and noodles, ice creams and chips. Akin to our results, a study conducted in Sao Paulo using 24-h recall showed that commonly consumed ultra-processed foods were carbonated beverages (31 %), biscuits (20 %), packaged snacks (16 %), processed meat (13 %) and ice cream (10 %) among adults^(38)^. Results from the present study showed that the intake of ultra-processed foods contributed to 17 % of total energy intake in the diets, which is lower compared with developed nations. Studies conducted using 24-h dietary recall showed that the intake of ultra-processed foods in developed nations contributed to 25 %, 42 % and 58 % of total energy intake in the diets in Korea, Australia and United States, respectively^(39,40)^. A study using the Euromonitor database showed that the sale of ultra-processed food in India was projected to rise from 2000 to 2017 with a greater percentage share from carbonated beverages, biscuits, sweet and savoury snacks^(41)^. Results showed that although nutrient intakes derived from ultra-processed foods were not in large amounts, for example, the total fat from ultra-processed food was 16 g, carbohydrate was 52 g, protein was 6·4 g, free sugar was 17 g (approximately 3 teaspoons) and Na was 266 mg; still, ultra-processed foods contributed to one-fifth of the total fat intake in the diet and around one-third of the free sugar and Na in the diets of Indian adults. Data from surveys conducted in urban India by the National Nutrition Monitoring Bureau in 2016 showed that ultra-processed foods contributed to 11·1 % of total energy intake, 10·3 % of protein, 10·7 % of carbohydrate and 11·6 % of total fat in the diet^(10)^. Over the years, the contribution of ultra-processed foods in the diets of urban Indians has increased, which is evident in the present study as these foods contributed to 17 % of total energy intake, 12 % of protein, 17 % of carbohydrate, 29 % of added sugar, 20 % of total fat and 33 % of Na intake in the diets among adults. Nutrition transition in India is witnessing a huge shift in dietary pattern as seen by an increasing intake of ultra-processed foods that have replaced home-cooked foods. Appropriate interventions at the right time can help to slow down an increase in the consumption pattern trajectory in lower middle-income countries such as India that presently matches with that of developed nations^(42)^. Overconsumption of ultra-processed food high in fat, sugar and salt is associated with obesity and the risk of developing many diet-related non-communicable diseases such as diabetes mellitus, CHD and cancers in adults^(43)^. Owing to the harmful effects of ultra-processed foods and increased prevalence of diet-related non-communicable diseases in India, efforts should be made to monitor and decrease the daily consumption of ultra-processed foods among Indian adults. In the present study, there were positive significant associations between the percent energy contributed by ultra-processed food and the risk of niacin and folate inadequacies. This means that with an increase in intake of calories from ultra-processed food the risk of nutrient inadequacy also increased among participants. In another study conducted in Mexico, nutrient intakes of vitamin B_6_, Ca, Zn, niacin and folate from ultra-processed food component of the diet were lower (*P* < 0·01) when compared with the non-ultra-processed food intake^(44)^. In the present study, some significant but weak negative correlations were observed in the case of Fe, vitamin A and riboflavin. This may be related to the kind of ultra-processed food consumed by participants. Some of the ultra-processed foods may be fortified and not necessarily high in fat, salt and sugar. A study conducted in Australia where fortified breads and breakfast cereals were excluded from the diet showed significant reductions (*P* < 0·05) in carbohydrate, fibre, thiamine, niacin, Fe and Zn intake in the diets of adults. Fulfilling these nutrient deficits may not be realistic from other cooked sources since there are barriers such as lack of time, lack of cooking skills and busy work schedules. Thus, healthfulness of ultra-processed foods should be assessed after considering the beneficial and harmful nutrients^(45)^. Some of the ultra-processed foods such as beverages and instant pre-mixes contain added micronutrients and vegetables to enhance the healthfulness of the food product^(46)^.

Advocating for reformulation of ultra-processed packaged foods to reduce the content of fat, sugar and salt in it will ensure healthier intakes among population^(47)^. Application of front-of-pack labelling schemes on ultra-processed foods can be effective to help consumers judge the healthfulness of a food product^(48)^. Policies such as taxation on ultra-processed food that are high in fat, sugar and salt should be implemented by governments to reduce their consumption^(49)^. A study conducted in Chile showed that fiscal policies such as the combined effects of applying an 18 % tax on foods such as sweets and snacks along with subsidies on fruits and vegetables by removing the 19 % value-added tax is a way to improve dietary choices even among those in the highest income quintiles^(50)^.

The strength of the present study is that the results can be used as baseline information before starting an intervention to improve diet quality. This study also measures the risk of nutrient inadequacy using the latest ICMR Nutrient requirements (2020) and links it with the proportion of total energy contributed by ultra-processed food in the diet. The limitation of the present study was that the study was conducted in Delhi on only upper middle-income and high-income group young adults, which limits generalisation of the findings to other regions of the country, income and age categories. In the case of collecting dietary intakes through 24-h dietary recall, a recall bias cannot be ruled out. However, an effort to reduce the bias was made by displaying three dimensional models of spoons and bowls, along with their respective picture cards, which helped by acting as memory cues.

## Conclusion

Reduced dietary diversity and food group adequacy can still exist irrespective of the income status of an individual. To improve the micronutrient adequacy and dietary diversity, investment in behaviour change communication strategies can be rewarding. It is important to generate knowledge and understand the perceptions of the community regarding healthy eating to address the problem of micronutrient inadequacies in diets. Policymakers should monitor the consumption of ultra-processed foods in India and take appropriate measures to improve dietary habits. It is also important for the industry to reformulate ultra-processed foods to decrease fat, salt and sugar content and offer minimally processed, micronutrient-rich food products to the consumer.

## Supporting information

Mediratta et al. supplementary material Mediratta et al. supplementary material

## Supplementary material

For supplementary material accompanying this paper visit https://doi.org/10.1017/S1368980023002112
