# Supplementary material for: Intake of ultra-processed food, dietary diversity and the risk of nutritional inadequacy among adults in India
Source: Public Health Nutr. 2023 Oct 2;26(12):2849–58. doi: 10.1017/S1368980023002112 (PMC10755415; doi:10.1017/S1368980023002112)
Supplement: Mediratta et al. supplementary material [file S1368980023002112sup001.docx]

**Supplementary file**

**Table 1: Sample size estimation**

| Sample Size for males = 293  Population size (for finite correction factor or fpc) (N) = 1000000  Hypothesized % frequency of outcome factor in the population (p)= 74.4%±5  Confidence limits as % of 100 (d)=5%  Design effect (DEFF)=1  Confidence Level (%)=95% |
| --- |
| Sample Size for females = 296  Population size (for finite correction factor or fpc) (N) = 1000000  Hypothesized % frequency of outcome factor in the population (p)= 74%±5  Confidence limits as % of 100 (d)=5%  Design effect (DEFF)=1  Confidence Level (%)=95% |
| Total sample N **=** 293+296= 589 |
| Taking into account 10% attrition rate, in total 684 participants were enrolled, however only 589 participants completed the study. |

The equation used to calculate the sample size for cross sectional study:

n = [DEFF*Np(1-p)]/ [(d2/Z21-α/2*(N-1)+p*(1-p)]

**Table 2: Statistical codes used for calculation of probability risk of inadequacy**

| **#Normal Intake and normal requirement#**   1. muR<-EAR ;sigR<-(RDA-EAR)/1.96 2. muIntake<-mean(Intake, na.rm=T) 3. sigIntake<-sd(Intake, na.rm=T) 4. riskN<- function(x, mu,sig) {1- pnorm(x, mu, sig)}   risk(x)<-function(x){1-pnorm(x,mean = muR, sd = sigR)}   1. y<-rnorm(n=100000, mean= muIntake,sd = sigIntake) 2. PIA<-mean(risk(y)) |
| --- |
| **#Log Normal Intake and normal requirement#**   1. muR<-EAR ;sigR<-(RDA-EAR)/1.96 2. nutrientIntake<- nutrientIntake [nutrientIntake >0] 3. muIntake<-mean (log(nutrientIntake), na.rm=T) 4. sigIntake<-sd(log(nutrientIntake),na.rm=T) 5. risk(x)<-function(x){1-pnorm(x,mean = muR, sd = sigR)}   riskN<- function(x, mu,sig) {1- pnorm(x, mu, sig)}   1. y<-rlnorm(n=100000, meanlog = muIntake,sdlog = sigIntake) 2. PIA<-mean(risk(y)) |
| **#Log Normal Intake and log normal requirement#**   1. muR<-log(EAR);sigR<-(log(RDA)-log(EAR))/1.96 2. nutrientIntake <- nutrientIntake [nutrientIntake >0] 3. muIntake<-mean (log(nutrientIntake), na.rm=T) 4. sigIntake<-sd(log(nutrientIntake),na.rm=T) 5. risk(x)<-function(x){1-plnorm(x,meanlog = muR, sdlog = sigR)}   riskLN<- function(x,logmu, logsig) {1 - plnorm(x,logmu, logsig)}   1. y<-rlnorm(n=100000, meanlog = muIntake, sdlog = sigIntake) 2. PIA<-mean(risk(y)) |
